# Palmatine Inhibits the Pathogenicity of *Aeromonas hydrophila* by Reducing Aerolysin Expression

**DOI:** 10.3390/foods11203250

**Published:** 2022-10-18

**Authors:** Jing Dong, Tianhui Yan, Qiuhong Yang, Yi Song, Bo Cheng, Shun Zhou, Yongtao Liu, Xiaohui Ai

**Affiliations:** 1 Yangtze River Fisheries Research Institute, Chinese Academy of Fishery Sciences, Wuhan 430223, China; 2 College of Food Science and Engineering, Bohai University, Jinzhou 121013, China; 3 Chinese Academy of Fishery Sciences, Beijing 100039, China

**Keywords:** *Aeromonas hydrophila*, aerolysin, palmatine, anti-virulence, antibiotic resistance

## Abstract

*Aeromonas hydrophila*, an opportunistic aquatic pathogen widely spread in aquatic environments, is responsible for a number of infectious diseases in freshwater aquaculture. In addition, *A. hydrophila* can transmit from diseased fish to humans and results in health problems. The occurrence of antibiotic-resistant bacterial strains restricts the application of antibiotics and is responsible for failure of the treatment. Moreover, residues of antibiotics in aquatic products often threaten the quality and safety. Therefore, alternative strategies are called to deal with infections caused by antibiotic-resistant bacteria. Aerolysin, one of the most important virulence factors of *A. hydrophila*, is adopted as a unique anti-virulence target on the basis of the anti-virulence strategy to battling infections caused by *A. hydrophila*. Palmatine, an isoquinoline alkaloid from a variety of herbal medicines that showed no anti-*A. hydrophila* activity, could reduce hemolysis of the bacterium by decreasing aerolysin production. The results of the qPCR assay demonstrated that the transcription of the *aerA* gene was suppressed. Moreover, cell viability and in vivo study showed that palmatine treatment could decrease the pathogenicity of *A. hydrophila* both in vitro and in vivo. In summary, palmatine is a leading compound against *A. hydrophila*-associated infection in aquaculture by inhibiting the expression of aerolysin.

## 1. Introduction

Aquatic products, supplying over 8% animal protein to human diet, play critical roles in nutrition and food security worldwide, particular in developing countries [1,2]. Therefore, aquaculture has become the fastest growing industry in food production all over the world [3]. The rapid development of aquaculture results in the outbreak of infectious diseases. Among all the bacterial pathogens, *Aeromonas hydrophila* (*A. hydrophila*) is the main cause of infectious diseases in freshwater aquaculture systems [4]. Infections caused by the bacterium often result in severe mortality worldwide [5]. Moreover, *A. hydrophila* has been considered to be an emergent foodborne pathogen associated with a variety of diseases as it is transmitted from aquatic foods or water to humans [6]. The main approaches dealing with diseases caused by pathogenic bacteria are vaccines and antibiotics. Due to the limitations of aquatic vaccines, achievements have not been made in aquaculture [7]. However, indiscriminate use of antibiotics causes a number of problems, such as antibiotic resistance and residues of antibiotics in aquatic products and in the aquatic ecosystem. Antibiotic-resistant *A. hydrophila* strains have been widely isolated, exhibiting a high resistance rate and a broad resistance spectrum [8,9]. The increasing incidence of resistant strains limits the treatment of *A. hydrophila* infections in aquaculture. Consequently, novel drugs are needed in the battle against drug-resistant *A. hydrophila* infections. 

Due to the current shortages of antibiotic drugs and the decline of development of novel antibiotics, alternative strategies and novel treatment options are needed both in aquaculture and human medicine [10,11]. With the increasing knowledge of molecular pathways of bacterial infections and roles of bacterial virulence factors, strategies targeting to virulence factors provide novel opportunities to overcome the challenges of resistant bacterial infections [11]. Aerolysin is secreted as a water-soluble inactive precursor of 52 KDa named proaerolysin [12,13]. After cleaving the 43-residue C-terminal peptide, proaerolysin is activated, which exhibits cytolytic activity [14,15]. Aerolysin can form a heptamer on target cells with transmembrane channels and disrupt cells [16,17,18]. A previous study demonstrated that aerolysin is essential for the establishment and subsequent maintenance of infection for *A. hydrophila* by disrupting the aerolysin gene [13]. Injection of aerolysin could cause mortality in animal models with similar mode of action of live bacteria injection, indicating that aerolysin could be developed as a novel anti-virulence target dealing with *A. hydrophila-*associated infections [17]. 

**Figure 1:**
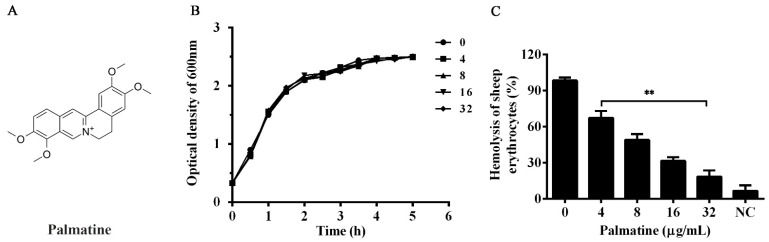
Inhibitory effect of palmatine on hemolysis induced by aerolysin in bacterial supernatants. (**A**) Chemical structure of palmatine. (**B**) Growth curves of *A. hydrophila* XS-91-4-1 co-cultured with different concentrations of palmatine. Values of each data point are the averages of three independent experiments. (**C**) Hemolytic activity of *A. hydrophila* culture supernatants treated with increasing concentrations of palmatine. Hemolysis of 1% Triton-X-100-treated sheep red blood cells served as the positive control, whereas hemolysis of bacterial supernatant without trypsin treatment served as the negative control (NC). Values represent the mean and standard deviation of three independent assays. Statistical significance was determined by Student’s *t*-test; ** indicates *p* < 0.01.

Phytotherapeutics with a number of biological activities have a long history in treating bacterial infections, both in human and animals, for centuries [19]. In aquaculture, phytotherapeutics have been considered as the route screening drugs against bacterial diseases, replacing antibiotics, and have been well-studied in recent years [20]. Moreover, herbal medicines can decrease the environmental risks and residues in animal tissues through the use of antibiotics [21]. Thus, herbal medicines have become promising candidates in aquaculture. Palmatine (Figure 1A) is an isoquinoline alkaloid that can be isolated from a number of traditional Chinese medicines. A range of pharmacological effects of palmatine have previously been reported, such as anti-bacterial, anti-inflammatory, anti-viral, and anti-cancer effects [22,23]. Palmatine has antibacterial effects against *Clostridium perfringens*, *Bifidobacterium bifidum*, *Helicobacter pylori*, and several other bacteria in vitro [23,24]. This study aims to identify an inhibitor alternating the use of antibiotics in aquaculture against the pathogenicity of *A. hydrophila*. As desired, we found that palmatine could provide significant protection for channel catfish infected with *A. hydrophila* by suppressing the transcription of the aerolysin-encoding gene *aerA*.

## 2. Materials and Methods

### 2.1. Bacterial Strains and Regents

*A. hydrophila* strain XS-91-4-1 (CCTCC AB 208146) was stored in our lab. Palmatine was obtained from Chengdu Must Bio-Technology Co., Ltd. (Chengdu, China) and enrofloxacin was purchased from the National Institutes for Food and Drug Control (Beijing, China). DMSO was used to prepare palmatine and enrofloxacin stock solutions for in vitro studies. For experimental therapeutic study, palmatine was suspended in 10% Tween 80–saline buffer. 

### 2.2. Determination of MICs

A susceptibility assay was carried out by the micro-dilution method in 96-well cell plates (Corning, New York, NY, USA) containing 200 μL MHB medium (Hopebiol, Qingdao, China) in each well, according to the guidance of CLSI [25]. Briefly, palmatine and enrofloxacin were diluted by MHB medium with a volume of 100 μL in 96-well plates. The concentrations for palmatine and enrofloxacin ranged from 256 to 0.5 μg/mL and 32 to 0.03125 μg/mL, respectively. Bacterial cells cultured in LB medium were acquired to about 5 × 10^5^ CFU/mL. Then, 100 μL of cells was added and they were further cultured for 18–20 h. MICs were defined as the lowest concentrations without visible growth. 

### 2.3. Growth Curves

Growth curve assay was carried out according to previously reported protocols [26]. Briefly, bacterial culture incubated overnight was inoculated into fresh LB medium of 100 mL and cultured to OD_600nm_ of 0.3; then, 10 mL of the suspension was equally divided into 5 glass flasks. Following the addition of palmatine at certain concentrations, the mixtures were further incubated for 5 h. The absorption at 600 nm was determined to monitor the bacterial growth with different concentrations of palmatine using a visible spectrophotometer (VWR, Conshohocken, PA, USA) every 30 min. 

### 2.4. Hemolytic Assay

Hemolytic activity assay was performed according to a previous study with some modifications [27]. XS-91-4-1 was incubated at 28 °C to OD_600nm_ of 0.3; then, palmatine at various concentrations was added after the suspension was divided into 5 flasks. DMSO served as the drug-free control. Centrifugation was used to septate bacterial supernatants when the value of OD_600nm_ in each flask reached 1.5. After sterilization by 0.22 μm filters, aerolysin in the cultures was activated by the addition of trypsin. For hemolytic activity assays, the hemolytic system contained 875 μL hemolysis solution, 100 μL trypsin-treated supernatant, and 25 μL freshly washed erythrocytes. After an incubation at 37 °C for 20 min without shaking, unlyzed erythrocytes were removed by centrifugation, and OD_543nm_ was measured. We used 0.1% Triton X-100 to make 100% hemolysis control, while the supernatant of the drug-free group without trypsin treatment served as the negative control. 

### 2.5. Western Blotting Assay

Bacterial supernatants collected above were employed as samples for Western blotting assay, according to a previous study [28]. The number of total proteins in the supernatants was evaluated by a commercial bicinchoninic acid protein assay kit (Thermo Fisher Scientific, Waltham, MA, USA) before sampling. After electrophoresis, proteins in the gel were then transferred to a polyvinylidene difluoride (PVDF) membrane and then incubated for 2 h at room temperature with a primary anti-aerolysin polyclonal antibody (prepared in our laboratory) at a concentration of 1:1000 after blocking with 5% nonfat milk in TBST. The membrane was washed and hatched with a goat anti-rabbit IgG conjugated with HRP. An ultra-high sensitivity ECL kit was used to detect aerolysin levels in supernatants with a 5 min incubation and pictured by a ChemiDoc XRS+ imager.

### 2.6. qPCR

Bacterial cells collected in a hemolysis assay were used to separate total RNA using a commercial RNA extraction kit. DNase I was added to remove remaining DNA, and the concentrations of each sample were determined by a micro-volume spectrophotometer. qPCR reaction was carried out after cDNA synthesis. The amplification system contained 12.5 μL 2 × SYBR Premix Ex Taq, 1 μL cDNA, and 10 μM primers. The reaction parameters were as follows: initial step of hold at 95 °C for 30 s, then 40 cycles of denaturation at 95 °C for 5 s and annealing at 56 °C for 30 s. PCR products were detected by the increasing in fluorescence by SYBR Green attached to amplified DNA, and Ct values were calculated. Primers for detecting *aerA* were 5′-TCTACCACCACCTCCCTGTC-3′ (forward) and 5-GACGAAGGTGTGGTTCCAGT-3′ (reverse), while 5′-TAATACCGCATACGCCCTAC-3′ (forward) and 5-ACCGTGTCTCAGTTCCAGTG-3′ (reverse). We employed 16s rRNA as the internal housekeeping gene. The levels of transcription after treatment with palmatine were calculated by the 2^-ΔΔCt^ method according to a previous study [29].

### 2.7. Cytotoxicity Assay

Cytotoxicity assay was conducted according to previously published protocols with some modification [27]. Briefly, A549 cells were incubated in DMEM containing 10% fetal bovine serum, 100 units/mL penicillin, and 100 μg/mL streptomycin at 37 °C with 5% CO_2_. Cells were trypsinized and added into a 96-well microplate at a concentration of 1.5 × 10^5^ cells per well for an incubation at 37 °C overnight before use. For lactate dehydrogenase (LDH) release assay, cells were cultured in DMEM without FBS after being washed with sterile phosphate-buffered saline (PBS). Then, cells were co-cultured with bacterial supernatants obtained from bacterial culture after treatment with palmatine for 1 h at 37 °C. We collected 120 μL cell supernatants and moved them to a new plate after centrifugation. LDH release regent (Yeasen, Shanghai, China) mixture was added into cell supernatants and maintained for further 1 h. After incubation, the OD _490nm_ values were determined in each well using a microplate reader. Cells without any treatment were represented as negative control; cells co-cultured with 1% Triton X-100 were defined as 100% LDH release control. Cells treated with bacterial supernatants as described above were washed thrice, and then live/dead staining was performed by labelling the cells with calcein AM and propidium iodide mixture (Yeasen, Shanghai, China). A fluorescence microscope (490 nm excitation) was used to acquire the images.

### 2.8. Animal Studies

In vivo studies were performed in our breeding center in accordance with, and with the approval of, the Animal Welfare and Research Ethics Committee. Channel catfish (100 ± 5 g) were chosen as experimental animals to establish the infection model [28]. A total of 90 fish were divided into 3 groups and maintained in tanks for one week at 28 °C with flow-through water system. *A. hydrophila* XS-91-4-1 at mid-logarithmic phase was harvested by centrifugation and washed with PBS. Then, bacterial cells were re-suspended, and a density of 1 × 10^8^ CFU/mL by McFarland standards was produced. After being anaesthetized by tricaine methanesulphonate (MS-222, 250 μg/mL), 200 μL bacterial suspension was injected into fish in the positive group and the palmatine treating group intraperitoneally to establish the infection model, while the negative control group was given sterile PBS. Then, 50 mg/kg palmatine was administered to fish in the treated group 6 h post-infection. Positive and negative control groups were given 10% Tween 80-saline buffer. The course of treatment was sustained for 3 days in 12 h intervals. The mortalities were recorded for a further 5 days, and the mortality was calculated. 

### 2.9. Statistical Analysis

Differences in hemolytic activity, qPCR, and LDH release assays were calculated using Student’s *t*-test by SPSS 14.0. Means ± SD were used to express the data. Kaplan–Meier estimates and log-rank test were used to determine the significance of mortality of different treatments. 

## 3. Results

### 3.1. Palmatine Had No Role on the Growth of A. hydrophila

Enrofloxacin, one of the most frequently used antibiotics for treating bacterial infections of aquaculture in China, was chosen as a control to evaluate the susceptibility of the strain. The MIC value of palmatine was higher than 256 μg/mL, while 4 μg/mL was the amount for enrofloxacin. The results revealed that palmatine had no anti-*A. hydrophila* activity, while the bacterial strain used here was resistant to enrofloxacin. Moreover, similar results were observed according to the growth curve assay. No influence was found on bacterial growth by palmatine at concentrations of 0, 4, 8, 16, and 32 μg/mL in 5 h (Figure 1B). The impact of DMSO was further determined by the addition of DMSO to the drug-free group, and the results showed that DMSO in both assays had no influence on the growth of *A. hydrophila*. These results illustrated that palmatine would not affect bacterial growth, indicating that weaker selective pressure would be given by palmatine than antibiotics. 

### 3.2. Palmatine Decreased Hemolysis of A. hydrophila Induced by Aerolysin

**Figure 2:**
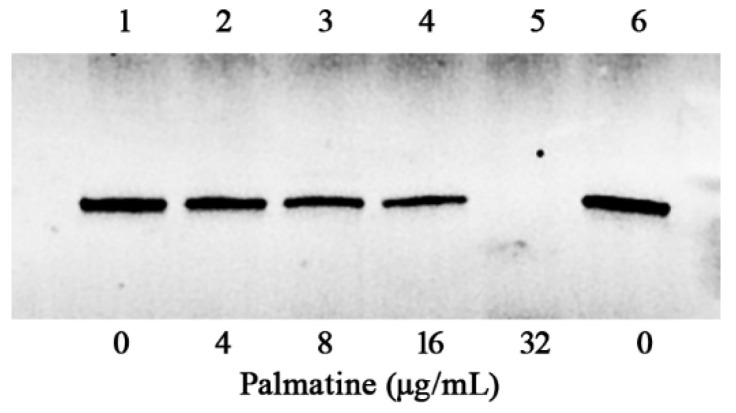
Western blot assay of aerolysin production in the supernatants after they were co-cultured with the indicated concentrations of palmatine.

Hemolysis mediated by aerolysin secreted by *A. hydrophila* in supernatants was declined dose-dependently after the co-incubation with palmatine at various concentrations (Figure 1C). Percent hemolysis reduced to 67.22 ± 5.78, 48.90 ± 4.93, 31.53 ± 3.06, and 18.48 ± 5.17% when the concentrations of palmatine reached 4, 8, 16, and 32 μg/mL, respectively, while 98.33 ± 2.50% was the case for drug-free supernatant. Compared with palmatine-free control, statistical significance of hemolytic activities was observed in the groups with palmatine at the concentrations of 4 μg/mL and above. Supernatant without trypsin treatment was served as a negative control (NC), showing only little hemolytic activity (Figure 1C). The findings indicated that the main course of hemolysis under our experimental conditions was the secretion of aerolysin to bacterial cultures. The results above indicated that bacteria co-cultured with different concentrations of palmatine affected the production or activity of aerolysin. Hemolytic activity using purified aerolysin with different concentrations of palmatine was performed, and no inhibitory effect was observed. To clarify the impact of palmatine on aerolysin production, Western blot was then conducted. As expected, the amount of aerolysin was reduced following the increasing concentrations of palmatine (Figure 2). When palmatine reached 32 μg/mL, no visible aerolysin was detected by Western blot. Taken together, the findings indicated that palmatine could decrease aerolysin-induced hemolysis by inhibiting aerolysin production.

### 3.3. Quantitative Real-Time PCR Results

**Figure 3:**
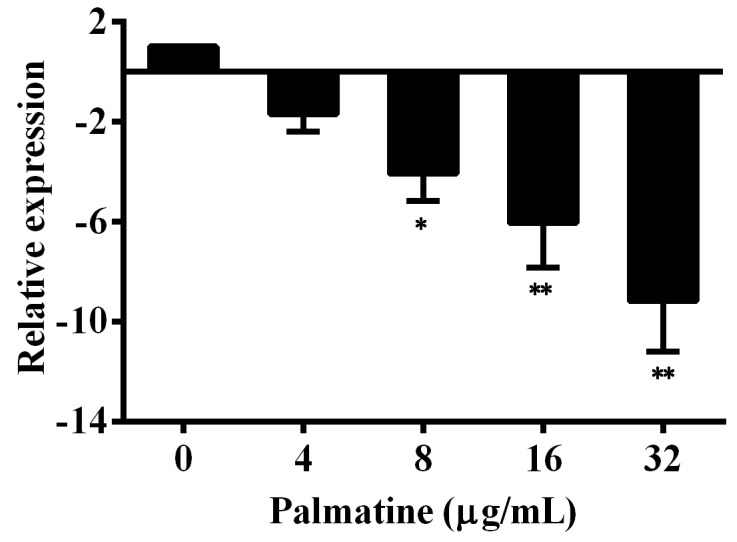
Impact of palmatine on the transcription of aerolysin-encoding gene *aerA*. Three independent assays were performed, and data are expressed as the mean ± SD; * indicates *p* < 0.05, ** indicates *p* < 0.01.

The results of hemolysis and Western blot assays demonstrated that *A. hydrophila* co-cultured with palmatine could decrease the production of aerolysin in bacterial supernatants, indicating that palmatine might inhibit the transcription of the encoding gene of aerolysin. Therefore, the total RNA was isolated to conduct the qPCR assay and determine the influence of palmatine on the *aerA* gene. As expected, the transcription of *aerA* gene was downregulated after it was co-cultured with different concentrations of palmatine in a dose-dependent manner (Figure 3). We observed 1.70 ± 0.68-, 4.09 ± 1.07-, 6.07 ± 1.78-, and 9.18 ± 2.02-fold decreases for the co-incubation with palmatine at concentrations ranging from 4 to 32 μg/mL, respectively. The reduction was statistically significant when palmatine reached 8 μg/mL and above. These findings demonstrated that the downregulation of the *aerA* gene resulted in the decrease in aerolysin after the co-incubation with palmatine at certain concentrations. 

### 3.4. Palmatine Reduced Aerolysin-Mediated A549 Cell Injury

**Figure 4:**
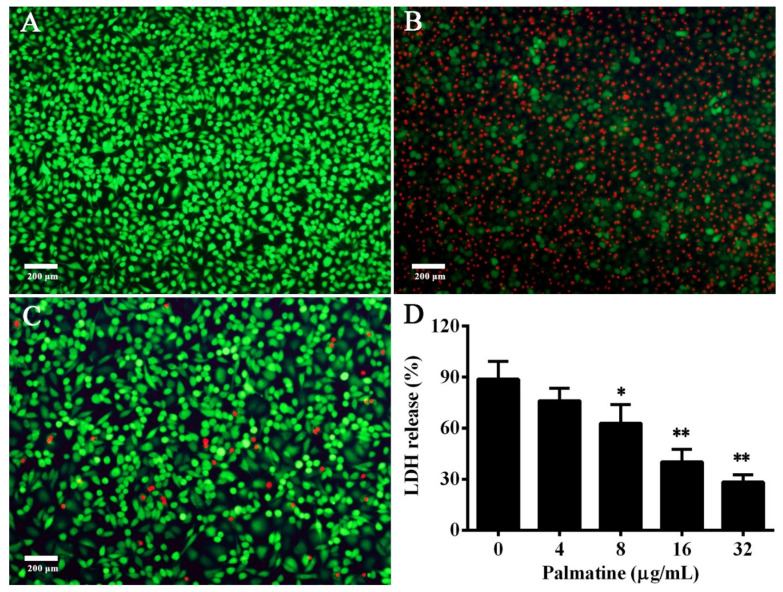
Palmatine reduced aerolysin-mediated cell injury. Cell viability was analyzed by live/dead staining regents, and pictures were captured by a fluorescence microscope; cell supernatants were used for evaluating cell injury by LDH release assay. (**A**) Untreated A549 cells. Most cells were alive and were stained with green. (**B**) Cells treated with bacterial supernatant without palmatine. Most cells were dead and were stained with red. (**C**) Cells treated with bacterial supernatant treated with 32 μg/mL palmatine. (**D**) LDH release of A549 cells after being co-cultured with bacterial supernatants treated with indicated concentrations of palmatine. Data of LDH release assay were the mean ± SD for three independent assays; * indicates *p* < 0.05, ** indicates *p* < 0.01.

Aerolysin can bind to a kind of eukaryotic cell and form β-barrel pores on the cell surface by inserting itself into the lipid bilayer, resulting in the destruction of cell membrane and osmotic lysis [30,31,32]. The toxin is sensitive to a variety of mammalian cells with glycosylphosphatidyl inositol (GPI)-anchored proteins, including epithelial cells and erythrocytes [33]. Thus, A549 cells were engaged to measure the protection of palmatine against cell damage caused by aerolysin. Cells in the 96-well plate after treatment with different supernatants were used for live/dead assay. Cells cultured in DMEM were distinguished by green fluorescence, indicating live cells (Figure 4A), while cells co-cultured with palmatine-free bacterial supernatant were stained by propidium iodide and exhibited red fluorescence, indicating dead cells (Figure 4B). As shown in Figure 4C, green cells and red cells were observed in cells treated with 32 μg/mL palmatine-treated supernatant, but most cells were shown in green in comparison with cells in the palmatine-free group. The finding showcased that significant protection could be provided by palmatine to A549 cells against *A. hydrophila* infection by decreasing the production of aerolysin. Moreover, cell injury after treatment with bacterial supernatants was determined by LDH release assay. LDH release of cells co-incubated with supernatants was reduced dose-dependently. The percentages of LDH release decreased to 76.05 ± 7.26, 62.89 ± 10.96, 40.30 ± 7.36, and 28.35 ± 4.24% when co-cultured with bacterial supernatants plus palmatine of 4, 8, 16, and 32 μg/mL, respectively, while the palmatine-free group was 88.79 ± 10.43%. The LDH release was statistically inhibited by adding palmatine of 8 μg/mL and above. LDH release to cell culture supernatants was a key feature of cells undergoing cellular damage [34]. Therefore, the results of LDH release revealed that palmatine could significantly inhibit the cell damage induced by *A. hydrophila*. Overall, the findings demonstrated that palmatine could alleviate cell injury caused by aerolysin. 

### 3.5. Results of In Vivo Study

**Figure 5:**
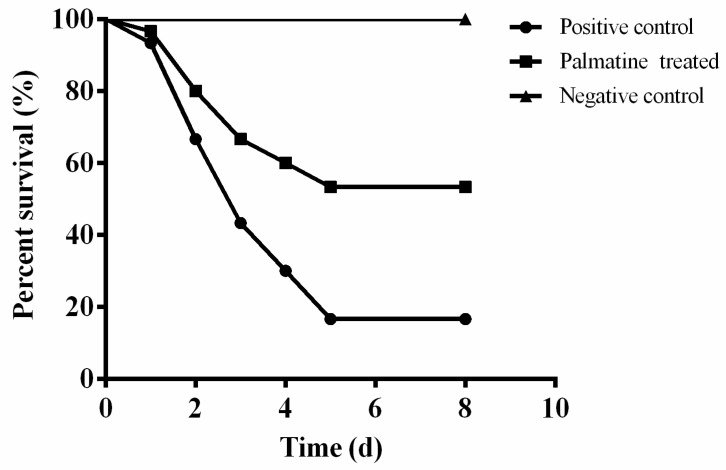
Palmatine treatment increased the survival rate of channel catfish challenged with *A. hydrophila*. Fish were administered with 50 mg/kg palmatine or sterile PBS at 12 h intervals for 3 days post-infection. Deaths were monitored for 8 days. The survival rate of the palmatine-treated group was significantly increased compared with infected fish without any treatment when analyzed by log rank test (*p* < 0.0001).

In vitro findings revealed that palmatine could inhibit aerolysin production of *A. hydrophila* and cover A549 cells from cell damage. The results indicated that a protective effect might have been provided by palmatine to channel catfish after *A. hydrophila* infection. Thus, the infection model was set up by challenging *A. hydrophila* with intraperitoneal injection. As shown in Figure 5, deaths were monitored of fish in positive and palmatine-treated groups 24 h after *A. hydrophila* infection. The percent survival of fish challenged with *A. hydrophila* without any treatment was 16.67%, while this was 53.33% of fish in the palmatine-treated group (Figure 5). All fish in the PBS-injected group were alive after an 8-day observation. The survival rate of palmatine-treated fish was remarkably improved compared with the *A. hydrophila* infection group. The results demonstrated that significant protection could be provided by palmatine to fish after *A. hydrophila* infection. 

## 4. Discussion

The discovery of antibiotics was one of the most important inventions in the 20th century that changed the therapeutic paradigm [35,36]. Nowadays, antibiotics are thoroughly used in prevention or treating diseases caused by pathogenic bacteria. Moreover, antibiotics have been introduced to livestock and fishery industries to deal with bacterial infections or as growth promoters [37]. In aquaculture, the frequency incidence of bacterial infections has resulted in the increasing use of antibiotics [38]. However, antibiotic resistance is emerged because antibiotics exert selective pressure to bacteria [39]. Additionally, antibiotic-resistant genes (ARGs) in aquatic environments have become a major source of ARGs in human and animal bacteria that threaten the health of humans. Therefore, novel strategies are needed to develop novel drugs overcoming the challenge caused by resistant bacteria in aquaculture. 

Several alternative strategies have been discovered for controlling bacterial infections in aquaculture, including phage therapy, inhibition of virulence, and quorum sensing disruption [40]. Pore-forming toxins (PFTs), secreted by a variety of pathogenic bacteria, have been reported with several biological activities [41]. Therefore, PFTs have already been identified as virulence factors in a number of important pathogens that can form pores after being inserted into cell membrane and result in cell death [42]. Therefore, PFTs are identified as unique targets for developing novel drugs fighting against drug-resistant bacteria [42]. Aerolysin belonging to the PFT family produced by *A. hydrophila* plays the essential function in the pathogenicity of *A. hydrophila* and is recognized as a marker of virulence in *A. hydrophila* [17]. Therefore, aerolysin has been employed as a target screening natural products via inhibiting the production of the toxin. *A. hydrophila* XS-91-4-1 used in the present study was initially isolated from diseased *Hypophthalmichthys molitrix,* and intraperitoneal injection of the bacterial cells could result in severe death in cultured fish [43]. Moreover, Zhu demonstrated that aerolysin is responsible for the virulence of the strain [44]. Our previous study found that natural compound could inhibit hemolysis induced by aerolysin of strains isolated from different sources [45]. Thus, XS-91-4-1 was used as a representative strain in the present study.

A number of compounds based on inhibiting activity, expression, or regulation of aerolysin have been identified. Eizo Takahashi et al. synthesized Indolo[3,2-b] quinolone derivatives and investigated the inhibitory effect against aerolysin activity in the supernatants of *A. hydrophila* and *Aeromonas sobria* strains. The results showed that the derivatives could directly neutralize the activity of aerolysin by hemolysis assay and were hopeful candidates in discovery of drugs by decreasing bacterial virulence [46]. However, some of the derivatives showed both anti-bacterial and anti-virulence bifunctional activities, indicating that the drug could provide selective pressure to bacteria and could result in resistance in the future. In the present study, palmatine, a natural alkaloid from herbal medicines, is similar to Indolo[3,2-b] quinolone in structure. However, the mechanism of palmatine against *A. hydrophila* infection was to decrease the production of aerolysin, which was absolutely different from Indolo[3,2-b] quinolone and its derivatives. A previous study found that magnolol could be used as an anti-virulence drug for treating fish infection challenged with *A. hydrophila* by reducing the expression of aerolysin, which offered a unique access in screening drugs from natural compounds against infections caused by antibiotic-resistant *A. hydrophila* [45]. However, the toxicity of magnolol might limit its application in aquaculture. Deng et al. determined the inhibition of palmatine against plant and animal bacterial pathogens, and the results demonstrated that palmatine had stronger activities against Gram-positive pathogens than Gram-negative pathogens [47]. Moreover, Xiao et al. found that palmatine could restrain the growth of *Microsporum canis* by destroying the cell membrane and organelles [48]. However, there was little knowledge on palmatine against *A. hydrophila*. According to the results of MIC, palmatine ranging from 0.25 to 256 μg/mL had no anti-*A. hydrophila* effect. The results revealed that palmatine could not affect bacterial growth of *A. hydrophila* at concentrations described above. Therefore, no selective pressure would be offered if treated with palmatine.

In previous studies, the roles of palmatine as anti-virulence agents against bacterial pathogens have been clarified. Wang et al. found that palmatine could inhibit the activity of plasmid-mediated quinolone resistance (PMQR) proteins QnrS and AAC(6′)-lb-cr, and could restore PMQR-mediated ciprofloxacin resistance of *Escherichia coli* [49]. Moreover, palmatine has been identified as a Sortase A inhibitor by binding to the catalytic center of the enzyme [50]. *N*o inhibitory effect against Gram-positive bacteria was observed, and therefore a lower selective pressure of inducing bacterial resistance was offered than those with anti-bacterial activity. Aghayan et al. demonstrated that palmatine could be used as an efflux pump inhibitor, increasing the susceptibility of antibiotics, which could reduce the emergence of antibiotic-resistant bacterial strains [51]. These findings indicated that palmatine was a potent anti-virulence agent by decreasing the pathogenicity or resistance of bacteria. In our present study, palmatine was developed as an inhibitor offering a notable protective effect to a fish infection model by control aerolysin expression levels. Different from resveratrol, palmatine had no impact against the quorum-sensing-mediated phenotype. Palmatine is the main chemical composition of traditional Chinese medicine (TCM) *Coptis chinensis Franch* and *Phellodendron amurense Rupr*. These medicines are the content of several aquatic drugs such as Huang Lian Jie Du San, Ban Lan Gen Da Huang San, and Pu Gan San, which are still extensively applied in freshwater aquaculture to deal with bacterial infections. In conclusion, palmatine could be chosen as a promising candidate in treating *A. hydrophila*-associated infections by inhibiting bacterial pathogenicity rather than inhibiting bacterial growth.

## Data Availability

The data presented in this study are available on request from the corresponding author.
